# The Levels of Leptin, Cystatin C, Neuropilin-1 and Tau Protein in Relation to Dietary Habits in Patients with Alzheimer’s Disease

**DOI:** 10.3390/jcm12216855

**Published:** 2023-10-30

**Authors:** Sylwia Bogdan, Anna Puścion-Jakubik, Katarzyna Klimiuk, Katarzyna Socha, Jan Kochanowicz, Ewa Gorodkiewicz

**Affiliations:** 1 Bioanalysis Laboratory, Faculty of Chemistry, University of Bialystok, Ciołkowskiego 1K, 15-245 Bialystok, Poland; sylwiabogdan93@gmail.com (S.B.); ewka@uwb.edu.pl (E.G.); 2 Department of Bromatology, Faculty of Pharmacy with the Division of Laboratory Medicine, Medical University of Białystok, Mickiewicza 2D Street, 15-222 Bialystok, Poland; katarzyna.socha@umb.edu.pl; 3 Podlasie Center of Psychogeriatrics, Swobodna 38 Street, 15-756 Bialystok, Poland; litwk@wp.pl; 4 Department of Neurology, Medical University of Białystok, M. Skłodowskiej-Curie 24a Street, 15-276 Bialystok, Poland; kochanowicz@vp.pl

**Keywords:** Alzheimer’s disease, diet, markers, ROC curve

## Abstract

Alzheimer’s disease (AD) is the most common form of dementia in older people. Its prevalence is expected to increase, and therefore it poses a serious challenge to the healthcare system. The aim of the study was to assess the concentration of leptin, cystatin C, neuropilin-1 and tau protein, as well as the influence of dietary habits on these parameters, in a group of AD patients (*n* = 110) compared to 60 healthy people (*n* = 60). It has been shown that AD patients, compared to healthy people, are characterized by significantly higher median concentrations of leptin (9.97 vs. 3.08), cystatin c (1.53 vs. 0.56) and tau protein (8.46 vs. 4.19), but significantly lower median neuropilin-1 (69.94 vs. 167.28). Multiple regression analyses showed that leptin levels could be explained by dietary habits in 27%, cystatin C in 51%, neuropilin-1 in 41% and tau protein in 25% of cases. Modification of eating habits may contribute to improving the values of the discussed parameters.

## 1. Introduction

Leptin is a hormone produced by fat cells that stimulates the appetite by sending information to the brain about the body’s energy resources. It works via the leptin receptors found mainly in the hypothalamus. Research suggests that its level is higher in females than in males.

There is mounting evidence from epidemiological studies that changes in body weight from obesity in middle age are associated with Alzheimer’s disease (AD), with weight loss occurring in the earliest stages of AD increasing the risk of developing AD [1]. 

Therefore, the factors that regulate body weight are likely to influence the development and progression of AD. Leptin, derived from adipocytes, has been shown to be a major body weight regulator, mainly by activating the nerve circuits of the hypothalamus. Leptin also has several pleiotropic effects, including regulating cognitive function and having neuroprotective effects, suggesting a potential link between leptin and AD [2].

Cystatin C (CysC) is a cystatin protein belonging to the cysteine proteinase inhibitors. CysC (or cystatin 3) is a protein encoded by the CST3 gene. All cells that contain a cell nucleus produce cystatin C (a chain of 120 amino acids). Originally, it was identified in human CSF, and subsequently it has been found in all body fluids and tissues [3]. 

Large amounts of CysC have been found in brain tissue, expressed by astrocytes, neurons and microglial cells [4]. Changes in serum levels of CysC have been observed in diseases such as chronic kidney disease, rheumatoid arthritis, cardiovascular diseases and neurological disorders [5]. One of the functions of CysC is to repair the nervous system, indicating a neuroprotective role [6]. It has been proven that polymorphism in the CysC gene causes a much greater risk of developing Alzheimer’s disease [7]. CysC was found together with beta-amyloid in amyloid plaques in the brains of Alzheimer’s patients [8,9,10].

Neuropilin-1 (NRP-1) is currently described in the literature as a key signaling node. It is a transmembrane receptor with a mass of 134 kDa [9]. It is expressed in the brain, placenta, heart and vascular system. It has been implicated as a biomarker of the inflammatory phenotype of microglia in damaged tissues in AD [11].

Tau protein is a key protein responsible for the assembly, stabilization and modulation of microtubules. Microtubules are very important for the proper functioning of neurons and the brain. In disease states, pathological modifications of the tau protein occur, resulting in, among other things, the aggregation of tau bubbles and the formation of paired helical filaments (PHFs) and neurofibrillary tangles (NFTs). These structures are a hallmark of Alzheimer’s disease and other tauopathies. Their accumulation results in cognitive impairment [12].

Therefore, the aim of our study was to assess the levels of CysC, leptin, neuropilin-1 and tau protein in people with AD, compared to healthy people, in order to identify which parameters may act as markers of this disease. Additionally, we assessed the importance of dietary habits in terms of their impact on the tested parameters.

## 2. Materials and Methods

### 2.1. Study Group

The study group included 110 patients (men: *n* = 30; women: *n* = 80) with early or moderate AD. They were patients of the Neurology Clinic of the Medical University of Bialystok (Poland) and the Podlasie Psychogeriatric Center in Bialystok (Poland). The patients’ clinical condition was assessed by a geriatrician according to the 1984 National Institute of Neurology, Communication Disorders and Stroke/Alzheimer’s and Related Disorders Association (NINCDS-ADRDA) criteria, updated in 2007 [13]. In order to assess the severity of the disease, the common scale of a mini-mental state examination (MMSE) was used, allowing a score of a maximum of 30 points. 

The patients ranged in age from 54 to 93 years. The control group consisted of 60 healthy people without any cognitive disorders, from 52 to 83 years. Detailed characteristics are presented in Table 1.

The exclusion criteria were autoimmune diseases, cancer and type 1 and type 2 diabetes.

The protocol of this study was approved by the Local Ethics Committee (consent number: R-I-002/210/2018). In addition, all participants gave written consent to participate in this study. 

### 2.2. Preparation of Blood Samples

The blood samples were collected using vacuum system tubes containing a clotting activator (Becton Dickinson, Grenoble, France). The samples were centrifuged for about 10 min at a temperature of approx 1000× *g*. The sera were collected and stored frozen (at −20 °C).

### 2.3. Determination of Leptin and Cystatin C Concentration

Leptin and CysC concentrations were measured using an SPRI biosensor. All the elements of biosensor preparation and optimization are described in detail in Matuszczak et al. and Sankiewicz et al.’s works [14,15]. The gold chips (glass plates with 1 nm titanium and 50 nm gold layers (SSens, Enschede, The Netherlands)) were manufactured as described in other papers [16,17]. The gold surface of the chip was covered with photopolymer and hydrophobic paint. The chips were rinsed with ethanol and water and dried under a stream of nitrogen. They were then immersed in 20 mM of cysteamine hydrochloride (Sigma, Steinheim, Germany) ethanolic solutions for at least 2 h, and after rinsing with ethanol and water, dried again under a stream of nitrogen. A rabbit monoclonal anti-leptin antibody (Abcam, Cambridge, UK) was immobilized on the thiol monolayer under suitable conditions. A similar procedure was performed for CysC ligand (receptor)–papain from papaya latex (Sigma, Steinheim, Germany).

The leptin-specific antibody (60 ng/mL) and papain solutions (1.5 µg/mL) in a PBS buffer were activated using NHS (250 mM) (Aldrich, Munich, Germany) and EDC (250 mM) (Sigma, Steinheim, Germany). Activation was carried out by adding the mixture of NHS and EDC (1:1) in a carbonate buffer (BIOMED, Lublin, Poland) solution (pH 8.5) into the antibody or papain solution, with vigorous stirring for 5 min at room temperature. 3 μL of this solution was placed onto active parts with amine-modified surfaces, and incubated at 37 °C for 1 h. After this time, the biosensor was rinsed with water. Next, the serum samples (5× diluted for leptin and 2× diluted for CysC with PBS buffer (BIOMED, Lublin, Poland)) were placed directly onto the prepared biosensor. The volume of the sample applied onto each measuring field was 3 μL. The time of the interaction with the antibody was a max of 10 min. The biosensor was washed with water and HBS-ES buffer solution pH = 7.4 (0.01 M HEPES, 0.15 M sodium chloride, 0.005% Tween 20, 3 mM EDTA) (BIOMED, Lublin, Poland) to remove unbound molecules from the surface. The leptin (Abcam, Cambridge, UK) concentration in the samples (after appropriate dilution) was read from a calibration curve prepared in the range of 0.1–3 ng/mL. For CysC (Sigma, Steinheim, Germany), also the concentration calibration curve was read in the range of 0.1–1 ng/mL.

Measurements using the SPRI method to determine the concentration of CysC and leptin were performed as described in previous studies, while the diagram of the apparatus is presented in [18]. As controls for the level of nonspecific binding, some of the parts of the biosensor covered with buffer were used. The SPRI signal was measured at a fixed SPR angle on the basis of registered images. A first image after immobilization of the antibody or papain was taken. Then, a second image after interaction with leptin and CysC was taken. The SPRI signal was obtained by subtracting the signal after and before interaction with a biomolecule, for each spot separately. The contrast values obtained for all pixels across a particular sample single spot were integrated. Then, the SPRI signal was integrated over the spot area. NIH Image J version 1.32 software was used to evaluate the SPRI images in 2D form and to convert the numerical signals into quantitative signals.

### 2.4. Determination the Concentration of Neuropilin-1

Preparation and measurement using the biosensor was performed as in the previous article [19]. First, the biosensor linker layer was immobilized to bind the antibody (receptor). The chip was then immersed in a 20 mM cysteamine solution for a minimum of 12 h at room temperature. After this time, the plate was removed and washed with a solution of anhydrous ethyl alcohol and water. In the next step, the mouse monoclonal antibody of human neuropilin-1 (R&D Systems, Minneapolis, MN, USA) was bound. For this purpose, 2–3 µL of an antibody solution of the appropriate concentration was applied to the cysteamine layer. Such a prepared chip was placed in an incubator for 1 h at 37 °C. In the next steps, the biosensor was removed and washed with HBS-ES and distilled water to remove excess unbound antibodies. To avoid non-specific adsorption, a BSA solution (C = 1 mg/mL) was applied to the biosensor surface and rinsed with distilled water. The biosensor prepared in this way captured the NRP-1 from the solution. About 2–3 µL of the analyzed solution (200 times diluted) was added dropwise to the active sites and left for 10 min. The biosensor was then rinsed with HBS-ES solution and water. The necessary measurements were carried out at pH = 7.40 (information contained in the reagents’ safety data sheets). For the NRP-1 (R&D Systems, Minneapolis, MN, USA), the concentration calibration curve was read in the range of 0.01–2.50 ng/mL.

The biosensor prepared in this way was placed on a prism in the SPRi device. Then, the appropriate angle of incidence of the laser light needed to carry out the measurements was set. The images were taken at a fixed SPR angle. First, an image of the antibody layer was recorded. In the next step, an image after interaction of the antibody with a solution containing neuropilin-1 was recorded. In the final step, the SPRi signal was calculated based on the difference between the signals obtained before and after the interaction of the antibody with the analyte. As a result of the binding of the analyte to the receptor, there was a change in the refractive index (RI), which was detected as changes in the intensity of the reflected light captured on the image from the CCD camera. Then, a mathematical analysis of the 2D image was performed using ImageJ software (NIH, Bethesda, MD, USA, version 1.8.0_172).

### 2.5. Determination of the Concentration of Tau Protein

The first step was to immerse the gold chip in a 20 mM cysteamine solution for a minimum of 12 h at room temperature. In the next steps, the chip was removed and washed with a solution of anhydrous ethyl alcohol and water. The next step was to bind to the linker the capture element (ligand) of the analyte from the test sample. We chose to use a rabbit monoclonal antibody (Abcam, Cambridge, UK) that had been previously activated with EDC/NHS.

On the cysteamine layer of the chip, 2–3 µL of the activated antibody solution at an appropriate concentration was dropped using a pipette. A properly prepared chip was placed in an incubator for 1 h at 37 °C. After this time, the chip was removed from the incubator and washed with HBS-ES and distilled water to remove excess antibody. To avoid non-specific adsorption, a BSA (Aldrich, Munich, Germany) solution (C = 1 mg/mL) was applied to one active part of the biosensor surface and rinsed with distilled water. About 2–3 µL of the analyzed solution (without dilution) was added dropwise to the active sites and left for 10 min. The biosensor was then rinsed with HBS-ES solution and water. The necessary measurements were carried out at pH = 7.40. The tau protein (Abcam, Cambridge, UK) concentration in the samples (after appropriate dilution) was read from a calibration curve prepared in the range of 3.35–15 pg/mL.

A biosensor was placed onto the prism in the SPRi device and the appropriate angle of incidence of the laser light was set. First, an image of the antibody layer was recorded. At the next stages, the interactions between the antibody and the solution containing our analyte were recorded. As a result of the binding of the analyte to the receptor (ligand), the refractive index (RI) changed, which was detected as changes in the intensity of the reflected light recorded on the image from the CCD camera. In the last step, the SPRi signal was calculated based on the difference of the signals obtained before and after the interaction of the antibody with the analyte. Mathematical analysis of the 2D image was then performed using ImageJ software (NIH, version 1.8.0_172) [20]. 

### 2.6. Assessment of Eating Habits

All participants of the study, independently or with the help of their caregivers, completed the Food Consumption Frequency Questionnaire (FFQ) developed by the Committee on Human Nutrition Sciences of the Polish Academy of Sciences. They assessed the frequency of consumption of 35 popular food groups—these were listed in detail in our earlier publication. Where respondents indicated consumption of products 12 to 30 times a month, this was referred to as “frequent”—the exception was fish (4 to 12 times/month is frequent consumption). If the products were consumed less frequently, they were classified as occasionally consumed [21].

### 2.7. Statistical Analysis

Data analysis was performed using Statistica v.13.3 software (TIBCO Software Inc., Palo Alto, CA, USA). The normality of the data distribution was checked using the Kolmogorov–Smirnov, Shapiro–Wilk and Lilliefors tests. A Mann–Whitney U test or Kruskal–Wallis ANOVA was used to assess the differences between the groups. The strength of the correlation was assessed using Spearman’s rank test. The influence of dietary habits on the level of the examined parameters was assessed using a stepwise multiple linear regression analysis. In addition, we performed ROC curve analysis to assess the diagnostic utility of the tested markers. For this purpose, we referred to the results from this publication, as well as to our previous research [22,23,24]. Differences at a significance level of *p* < 0.05 were considered significant.

## 3. Results

### 3.1. Levels of Leptin, Cystatin C, Neuropilin-1 and Tau Protein

The values of the examined parameters are characterized in Table 2. 

Leptin was the first tested compound. Its content significantly increases in AD patients compared to healthy subjects (9.97 vs. 3.08, *p* < 0.00001). Moreover, AD patients had significantly higher CysC levels (1.53 vs. 0.56, *p* < 0.00001). The level of tau protein also increases (8.46 vs. 4.19, *p* < 0.00001). In patients with this neurodegenerative disease, there is a significant decrease in the level of neuropilin-1 (69.94 vs. 167.28, *p* < 0.00001).

In addition, it was shown that gender and smoking did not affect the values of the examined parameters. We did not find any connections between the values of the examined parameters and the stage of disease advancement measured using the MMSE scale (*p* > 0.05) (Table 3 and Table 4).

### 3.2. The Influence of Eating Habits on Concentration of Study Parameters

The impact of eating habits on the levels of the factors studied in this project is presented in Table 5. A positive value of the β coefficient indicates that frequent consumption of a product/group of products increases the value of a parameter, while a negative value suggests the opposite effect. 

In the case of leptin, only two factors have a significant impact on its concentration (fruits increase its concentration and oils decrease it). The strength of the association is 27%. 

Consumption of fresh fish increases the level of CysC, while eating wholegrain bread, flour products and jams lowers the level. Stepwise linear regression analysis showed that dietary habits can influence the serum levels of this marker by 51%. 

We also showed that dietary habits can influence neuropilin-1 concentration by 41%. The concentration of this compound is increased by the consumption of sausage products and decreased by eating flour products and margarine. 

Regression analysis showed that the concentration of tau protein may depend in 25% on the patient’s eating habits—the consumption of butter and bread may reduce the value of this parameter.

### 3.3. Correlations between the Tested Parameters

In this publication, we assessed the strength of the relationship between the parameters studied in this project, as well as between these parameters and previously published data [21,22,23]. There is a strong correlation between the concentration of neuropilin-1 and MMP-1 (r = 0.49, *p* = 0.0001). It should also be emphasized that an increase in the concentration of tau protein is accompanied by a decrease in the concentration of selenium in serum (r = −0.23, *p* = 0.0186).

### 3.4. Diagnostic Value of the Tested Parameters

**Figure 1:**
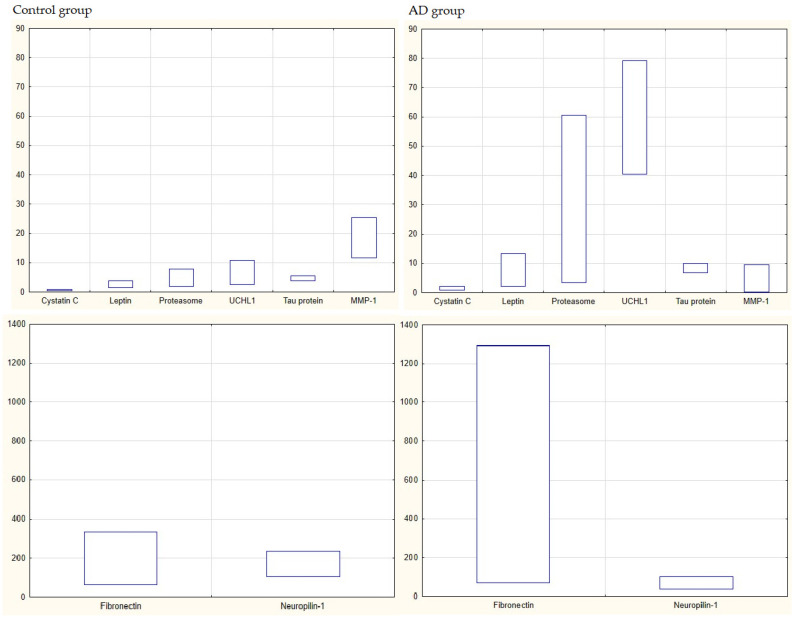
Ranges of values of tested parameters for healthy people and people with AD (units: cystatin C [µg/mL], fibronectin [µg/mL], Leptin [ng/mL], MMP-1 [ng/mL], neuropilin-1 [ng/mL], proteasome [µg/mL], tau protein [pg/mL], UCHL1 [ng/mL]). The term “proteasome” means a concentration of 20S.

The next stage of the analyses was to assess whether the ranges of values of the examined parameters were characteristic of AD patients. We assessed the factors studied in this work, but also those that were previously published by us [22,23,24]. Figure 1 shows the ranges of the minimum and maximum values of the tested compounds.

**Figure 2:**
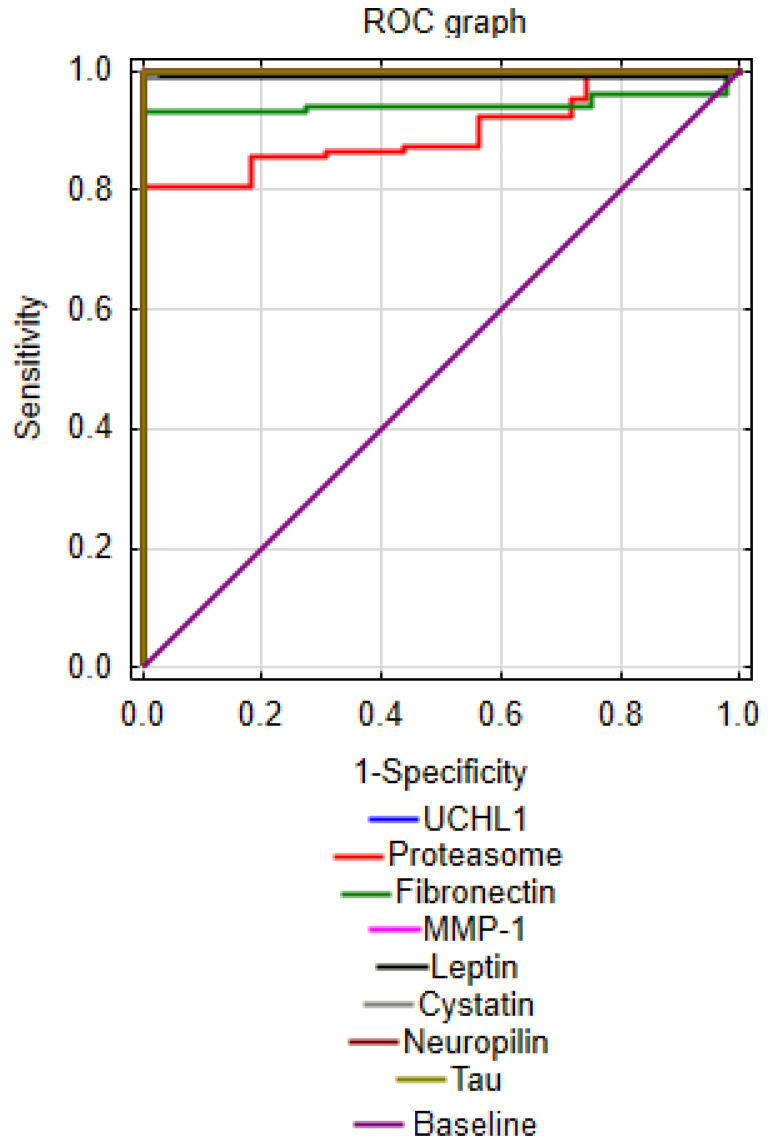
ROC graph for study markers of AD.

In order to indicate which parameters can be considered AD markers, ROC curves were also prepared—these are curves for assessing a classifier (factor) taking into account sensitivity and specificity. The performance of the indicator was expressed as the area under the ROC curve (AUC) with 95% confidence intervals (CIs) and p values. AUC values below 1 were calculated for those parameters for which the ranges of healthy people “mismatched” the ranges of sick people. In addition, the following parameters were calculated: cut-off, sensitivity, specificity, Youden’s index and positive and negative likelihood ratios. The cut-off values correspond to the highest accuracy of the markers (Table 6 and Figure 2).

The analyses performed showed that AD patients are characterized by high levels of UCHL1 compared to healthy people (from 40.52–79.16 to 2.45–10.81) and tau protein (from 6.84–9.91 to 3.75–5.56). In AD, there is a reduction in MMP-1 (range: 0.32 to 9.47 vs. 11.61 to 25.35) and neuropilin-1 (range: 36.44 to 99.24 vs. 105.68 to 233.96). In addition, the cut-off points for the values of the other parameters were indicated: proteasome concentration: 7.96; fibronectin: 346.14; leptin: 6.06 and CysC: 0.87.

## 4. Discussion

Contemporary research focuses on the search for markers of neurodegenerative diseases, as well as on effective methods of prevention and the improvement of cognitive functions. Therefore, we carried out a study whose aim was to assess the level of selected compounds in the serum of AD patients, as well as to determine what dietary habits may modify their concentration.

Our research has shown that patients with AD have higher levels of leptin compared to healthy individuals. Leptin is an adipokine that is secreted by adipose tissue and gastric mucosa. It has a central effect at the level of the hypothalamus, cerebral cortex or hippocampus. These regions express the long form of the leptin receptor LepRb, which is a unique receptor. It is capable of transmitting complete leptin signaling. The above areas are regions affected by chronic neurocognitive deficits such as AD or mild cognitive impairment [3]. Regression analysis showed that fat consumption may be a beneficial eating habit that lowers leptin levels. The aim of the study published by Kratz et al. (2002) was to assess the effect of fatty acid intake on serum leptin levels in healthy, non-obese subjects (*n* = 55; women: *n* = 25, men: *n* = 30). For 2 weeks, the participants were given a high-fat diet rich in saturated fat. They were then randomly assigned to one of three groups, receiving for 4 weeks refined olive oil (containing monounsaturated fatty acids), rapeseed oil (containing monounsaturated fatty acids and alpha-linolenic acid) or sunflower oil (containing n-6 polyunsaturated fatty acids). It was observed that olive oil and sunflower oil had no effect on the serum leptin levels. Canola oil contributed to a slight increase in leptin levels in men, but a large decrease in women. This indicates that the consumption of alpha-linolenic acid, present in, among other things, rapeseed oil, may modify leptin levels [25].

Regular consumption of good-quality fats is important because lipidomics analyses have indicated changes in lipid levels in the cerebrospinal fluid or brains of AD patients [26].

The results of our analysis indicate that fruits may have an unfavorable effect on leptin levels—this may be due to, among other things, their fructose content. Dai et al. (2006) [27] reported a reduced risk of AD, especially in the high-risk group, for people drinking juices, including fruit juices. This may be related to, among other things, the protective role of polyphenols.

However, it should be emphasized that weight loss is observed in AD, but it is mainly due to sarcopenia, not fat loss [28].

The second tested compound was CysC; it is considered a marker of, among other things, cardiovascular events and all-cause mortality. It is suggested that this is related to the nutritional status of the general population. For example, Zhang et al. (2023) [29] assessed that greater consumption of dairy products and meat and a greater variety of diet were associated with lower levels of CysC.

Elevated CysC levels in AD patients, similarly to our study, were indicated, among other contexts, in the Chinese population. The differences between healthy and sick people were less visible than in our Polish population (AD 1.034 ± 0.254 mg/L, control 1.010 ± 0.248 mg/L) [30]. 

The role of CysC in AD was initially explained by its colocalization with amyloid β in amyloid-loaded vessel walls and in the cores of senile amyloid plaques in the brains of AD patients. Moreover, CysC co-occurs with amyloid-β deposits in the brains of non-demented elderly people. CysC has been suggested to play a protective role in AD: CysC binds to Aβ and inhibits amyloid β oligomerization and fibril formation [31].

In our study, we showed that the level of CysC may be reduced by the consumption of, among other things, wholemeal bread. This is according to recently published research. Wang et al. (2023) [32] assessed the dietary habits of 2958 participants (mean age: 61 ± 9 years). The follow-up lasted an average of 12.6 years. There were 322 cases of dementia, including 247 people diagnosed with AD. People with the highest total intake of wholegrains were identified as having a lower risk of dementia from all causes, including AD, than those with lower intakes.

In our study, we observed a significant decrease in neuropilin-1 in AD patients. This is puzzling compared to the other literature’s data. For example, in a mouse model, the NRP-1 gene was shown to be frequently overexpressed in the AD model. The expression level varied depending on age and disease progression [33]. The analysis of eating habits showed that an increase in this parameter may be influenced by the consumption of sausages, and it is increased by the use of margarine. The main controversy surrounding the consumption of margarine stems from the presence of trans fats in it. These fats are incorporated into the brain cell membranes, changing the ability of neurons to communicate and reducing mental performance. Modern research also suggests the impact of trans fat consumption on increased risk of depression, as well as cognitive decline [34]. It should be emphasized that the presence of trans fatty acid isomers results from the partial hydrogenation of vegetable oils. Currently, the following are used: transesterification methods. The current legal requirements limit the content to 2 g/100 g, which does not pose a health risk [35].

The last component tested was tau protein. We demonstrated a significant increase in this parameter in AD patients. The deposition of tau aggregates is an important feature of AD. This results in the appearance of neurodegeneration and the manifestation of clinical symptoms [36]. 

Knowledge about the pathophysiology of AD has changed over the last several decades. It was based, among other things, on the “amyloid cascade hypothesis”, that is, that an increase in the level of amyloid-β is a key aspect in AD, which results in the pathology of the tau protein. This results in the death of neurons and is the beginning of AD. A new approach implicates the pathogenesis of AD, in which extracellular amyloid-β and tau oligomers act in parallel and upstream of the amyloid-β protein precursor (AβPP) [37]. Tau synthesized in the cerebral compartment can be released into the interstitial fluid, catabolized or retained in neurofibrillary tangles. Tau protein that is released into the interstitial fluid can mix with the cerebrospinal fluid and enter the plasma [38]. 

Data on butter consumption in AD are not clear. In our study, we showed that consuming butter can reduce the level of tau protein. Meanwhile, a study published by Berti et al. (2015) [39] presents a combination of ingredients “protecting against AD”: higher consumption of fresh vegetables and fruits, wholegrain products, fish and low-fat dairy products. It emphasizes the need to lower consumption of high-fat dairy products, processed meat, sweets, fried potatoes and just butter.

The literature’s data indicate that the levels of CysC and leptin may be influenced by environmental factors, but this aspect requires further analysis. For example, in people working with lead, CysC levels may be related to environmental exposure to thallium and its concentration in the urine [40]. Literature analyses (systematic reviews and meta-analyses) showed that leptin is a biomarker of stress; its level decreases after acute stress. Moreover, women show greater variability in leptin levels after stress, which indicates that leptin may have different effects on the development of obesity in response to stress depending on gender [41]. The reasons for women’s increased susceptibility to AD have not been sufficiently described, but based on the data presented, it can be assumed that one of the reasons may be sex hormones; they cause differential leptin biosynthesis in the stomach and different leptin concentrations in the serum of men and women [42].

Another important aspect is the fact that eating habits often change in people with AD, especially in advanced stages of the disease. Further research is needed on whether changes in diet caused by the disease (such as forgetting to eat, the need for third parties to prepare meals, changes in meal consistency preferences and changes in preferred products) may affect the level of biomarkers. Compared to various types of dementia, the pattern of changes in AD is not fully understood. It is indicated that swallowing problems develop at an early stage [43]. Previous research indicates that an accurate diagnosis of AD should be supported by clinical and pathophysiological tests, in vivo assessment of biomarker concentrations and memory tests. Typical markers include amyloid-β or tau protein [44]. Other markers include Aβ 1-42, P-Tau, T-Tau, neurogranin in cerebrospinal fluid, neurogranin and NFL occurring in neurodegeneration, IL-1B, IL-8, IL-33 and progranulin in inflammation. Down-regulation of miRNAs is indicated, including miR-125b, miR-181c and miR-26b. Up-regulation occurs in the case of miR-502-3p, miR-206 and miR-132 [45]. The newer markers of this disease include the level of Aβ42 in the plasma, the Aβ42/40 ratio and the neurofilament lumen [46]. Therefore, in the future, the issue of changes in the concentrations of selected compounds in the serum of AD patients should be considered according to the following aspect: whether they are specific biomarkers and appear in the early stage of the disease, or whether they may be a consequence of changes in dietary preferences and habits.

Dementia, neurodegenerative disorders and AD constitute such a significant challenge for modern medicine that the following years should provide an answer to the question of which parameters are the most specific and can be used in screening tests and routine diagnostics.

## 5. Conclusions

Patients with Alzheimer’s disease, compared to healthy people, are characterized by significantly higher levels of leptin, cystatin C and tau protein, and lower levels of neuropilin-1. One of the main components that determines their levels may be eating habits. Their modification may be an important element in the prevention of cognitive disorders.

## Figures and Tables

**Table 1:** Characteristics of the control and study groups.

Parameter	Control Group (*n* = 60) Av. ± SD Min.–Max.	Study Group (*n* = 110) Av. ± SD Min.–Max.
Gender	Male: 14, Female: 46	Male: 30, Female: 80
Age (years)	57.0 ± 7.9 52.0–83.0	77.8 ± 7.6 54.0–93.0
Body mass index (kg/m^2^)	nd	26.4 ± 4.1 17.8–40.2
MMSE	nd	20.4 ± 4.3 11–26
Smokes cigarettes	nd	Yes: 13, No: 97
Drinks alcohol	nd	Yes: 5, No: 105

Av.—average, MMSE—mini-mental state examination, nd—no data, SD—standard deviation.

**Table 2:** Concentration of selected protein substances in the AD group compared to the control group.

	Control Group	AD Group	*p*
Leptin [ng/mL]			
Av. ± SD	2.99 ± 0.58	9.90 ± 1.84	<0.00001
Min.–Max.	1.46–3.88	2.10–13.43	
Med.	3.08	9.97	
Q1–Q3	2.48–3.50	8.61–11.23	
Cystatin C [µg/mL]			
Av. ± SD	0.56 ± 0.10	1.53 ± 0.24	<0.00001
Min.–Max.	0.33–0.92	0.87–2.05	
Med.	0.56	1.53	
Q1–Q3	0.55–0.57	1.41–1.67	
Neuropilin-1 [ng/mL]			
Av. ± SD	168.77 ± 31.85	70.33 ± 16.08	<0.00001
Min.–Max.	105.68–233.96	36.94–99.24	
Med.	167.28	69.94	
Q1–Q3	139.99–192.48	57.76–85.10	
Tau protein [pg/mL]			
Av. ± SD	4.36 ± 0.45	8.38 ± 0.79	<0.00001
Min.–Max.	3.75–5.56	6.84–9.91	
Med.	4.19	8.46	
Q1–Q3	3.95–4.71	7.60–9.02	

Av.—average, Max.—maximum value, Med.—median, Min.—minimum value, Q1—lower quartile, Q3—upper quartile, SD—standard deviation.

**Table 3:** Concentration of selected protein substances depending on smoking and drinking alcohol (*p* > 0.05).

	Smokers	Non-Smokers	Alcohol Drinkers	Non-Drinkers
Leptin [ng/mL]				
Av. ± SD	9.41 ± 1.81	9.97 ± 1.78	10.11 ± 1.79	9.89 ± 1.79
Min.–Max.	7.12–12.26	2.10–13.43	8.16–12.03	2.10–13.43
Med.	9.62	9.97	9.97	9.97
Q1–Q3	8.12–10.61	8.94–11.18	8.56–11.81	8.90–10.98
Cystatin C [µg/mL]				
Av. ± SD	1.55 ± 0.24	1.52 ± 0.23	1.69 ± 0.18	1.51 ± 0.24
Min.–Max.	1.11–2.01	0.87–2.05	1.53–1.97	0.87–2.05
Med.	1.57	1.53	1.60	1.53
Q1–Q3	1.43–1.64	1.41–1.67	1.57–1.77	1.41–1.65
Neuropilin-1 [ng/mL]				
Av. ± SD	61.38 ± 15.82	71.51 ± 15.20	70.30 ± 2.80	70.31 ± 15.91
Min.–Max.	36.94–86.33	42.69–99.24	65.59–72.49	36.94–99.24
Med.	58.21	69.94	71.56	69.94
Q1–Q3	49.29–69.94	61.73–84.94	69.94–71.92	57.76–84.94
Tau protein [pg/mL]				
Av. ± SD	8.07 ± 0.86	8.43 ± 0.74	8.51 ± 0.78	8.38 ± 0.76
Min.–Max.	7.24–9.69	6.84–9.91	7.23–9.10	6.84–9.91
Med.	7.64	8.46	8.95	8.46
Q1–Q3	7.47–8.60	7.73–8.98	8.46–9.02	7.64–8.92

Av.—average, Max.—maximum value, Med.—median, Min.—minimum value, Q1—lower quartile, Q3—upper quartile, SD—standard deviation.

**Table 4:** Concentration of selected protein substances depending on gender and the result obtained in the MMSE test (*p* > 0.05).

	Women	Men	24–26 Points (MMSE Scale)	19–23 Points (MMSE Scale)	11–18 Points (MMSE Scale)
Leptin [ng/mL]					
Av. ± SD	9.88 ± 1.88	9.96 ± 1.52	10.20 ± 1.42	10.31 ± 1.83	9.43 ± 2.10
Min.–Max.	2.10–13.43	7.21–13.39	7.69–13.39	6.61–13.43	2.10–13.13
Med.	9.97	9.97	9.97	10.72	9.76
Q1–Q3	8.79–11.11	8.65–10.73	9.26–10.99	8.86–11.71	8.54–10.68
Cystatin C [µg/mL]					
Av. ± SD	1.53 ± 0.23	1.52 ± 0.25	1.57 ± 0.25	1.50 ± 0.23	1.46 ± 0.23
Min.–Max.	0.87–1.41	1.05–2.05	1.05–2.02	1.06–1.92	0.87–1.92
Med.	1.53	1.53	1.61	1.53	1.47
Q1–Q3	1.41–1.66	1.43–1.61	1.43–1.71	1.42–1.65	1.35–1.57
Neuropilin-1 [ng/mL]					
Av. ± SD	69.40 ± 16.09	72. 72 ± 14.00	74.51 ± 17.64	72.49 ± 14.68	66. 75 ± 17.17
Min.–Max.	36.94–99.24	43.67–98.78	42.69–98.78	42.29–95.55	36.94–99.24
Med.	69.83	69.94	77.09	69.94	69.75
Q1–Q3	57.76–84.32	67.06–86.33	67.40–87.25	66.32–87.02	54.80–82.34
Tau protein [pg/mL]					
Av. ± SD	8.36 ± 0.77	8.46 ± 0.75	8.64 ± 0.84	8.44 ± 0.75	8.33 ± 0.75
Min.–Max.	6.84–9.74	7.15–9.91	6.84–9.74	7.15–9.58	7.11–9.91
Med.	8.45	8.46	8.75	8.54	8.42
Q1–Q3	7.61–8.94	7.70–9.10	8.15–9.34	7.66–9.03	7.59–8.75

Av.—average, Max.—maximum value, Med.—median, Min.—minimum value, Q1—lower quartile, Q3—upper quartile, SD—standard deviation.

**Table 5:** Stepwise multiple linear regression analysis of the influence of the frequency of consuming food products on the concentration of study parameters.

Independent Variables	Β Coefficient (SE)	Significance Level	Adjusted R^2^
**Leptin**			
Fruit	**0.302 (0.124)**	**0.0184**	
Oils	**−0.280 (0.125)**	**0.0293**	0.27
Legumes	0.201 (0.123)	0.1075	
Tea	−0.223 (0.122)	0.0728	
Poultry	−0.148 (0.121)	0.2287	
Flour products	−0.147 (0.122)	0.2349	
Jam	−0.121 (0.126)	0.3411	
Margarine	−0.119 (0.118)	0.3157	
**Cystatin C**			
Fresh fish	**0.387 (0.119)**	**0.0022**	0.51
Wholemeal bread	**−0.320 (0.129)**	**0.0164**	
Flour products	**−0.307 (0.118)**	**0.0120**	
Jam	**−0.255 (0.119)**	**0.0377**	
Fruit	0.241 (0.124)	0.0579	
Honey	0.226 (0.140)	0.1136	
White cheese	0.206 (0.114)	0.0765	
Sweet drinks	0.193 (0.123)	0.1226	
Potatoes	0.178 (0.134)	0.1912	
Margarine	0.100 (0.134)	0.4569	
Sugar in beverages	0.074 (0.120)	0.5356	
Butter	−0.243 (0.138)	0.0850	
Boiled vegetables	−0.211 (0.112)	0.0655	
Sausage products	−0.183 (0.118)	0.128	
Poultry	−0.173 (0.111)	0.1246	
**Neuropilin-1**			
**Sausage products**	**0.247 (0.116)**	**0.0375**	0.41
**Flour products**	**−0.437 (0.116)**	**0.0004**	
**Margarine**	**−0.412 (0.122)**	**0.0014**	
Fruit	0.240 (0.125)	0.0602	
Wholemeal bread	−0.248 (0.134)	0.0688	
Honey	−0.213 (0.129)	0.1053	
White bread	−0.159 (0.129)	0.2229	
Potatoes	−0.140 (0.124)	0.2637	
**Tau**			
**Butter**	**−0.329 (0.116)**	**0.0063**	0.25
**White bread**	**−0.319 (0.115)**	**0.0073**	
Poultry	0.181 (0.119)	0.132	
Yellow and processed cheese	−0.218 (0.118)	0.0705	

Bold font was used for products with a statistically significant impact (*p* < 0.05).

**Table 6:** The diagnostic value of the analyzed parameters was assessed using ROC curves.

	AUC (95% CI)	SE	z	*p*	Cut-Off Point
Proteasome	0.901 (0.852–0.950)	0.025	16.071	0.0000	7.96
Fibronectin	0.945 (0.903–0.986)	0.021	20.992	0.0000	346.14
Leptin	0.991 (0.972–1)	0.009	51.95	0.0000	6.06
Cystatin C	1 (0.999–1)	0	1292.193	0.0000	0.87

## Data Availability

The data presented in this study are available on request from the corresponding author. The data are not publicly available for ethical and privacy reasons.
